# Large gyres as a shallow-water asymptotic solution of Euler’s equation in spherical coordinates

**DOI:** 10.1098/rspa.2017.0063

**Published:** 2017-04-12

**Authors:** A. Constantin, R. S. Johnson

**Affiliations:** 1 Faculty of Mathematics, University of Vienna, Oskar-Morgenstern-Platz 1, 1090 Vienna, Austria; 2 School of Mathematics and Statistics, Newcastle University, Newcastle upon Tyne NE1 7RU, UK

**Keywords:** Euler equations, spherical coordinates, gyres

## Abstract

Starting from the Euler equation expressed in a rotating frame in spherical coordinates, coupled with the equation of mass conservation and the appropriate boundary conditions, a thin-layer (i.e. shallow water) asymptotic approximation is developed. The analysis is driven by a single, overarching assumption based on the smallness of one parameter: the ratio of the average depth of the oceans to the radius of the Earth. Consistent with this, the magnitude of the vertical velocity component through the layer is necessarily much smaller than the horizontal components along the layer. A choice of the size of this speed ratio is made, which corresponds, roughly, to the observational data for gyres; thus the problem is characterized by, and reduced to an analysis based on, a single small parameter. The nonlinear leading-order problem retains all the rotational contributions of the moving frame, describing motion in a thin spherical shell. There are many solutions of this system, corresponding to different vorticities, all described by a novel vorticity equation: this couples the vorticity generated by the spin of the Earth with the underlying vorticity due to the movement of the oceans. Some explicit solutions are obtained, which exhibit gyre-like flows of any size; indeed, the technique developed here allows for many different choices of the flow field and of any suitable free-surface profile. We comment briefly on the next order problem, which provides the structure through the layer. Some observations about the new vorticity equation are given, and a brief indication of how these results can be extended is offered.

## 1. Introduction

**Figure 1.**
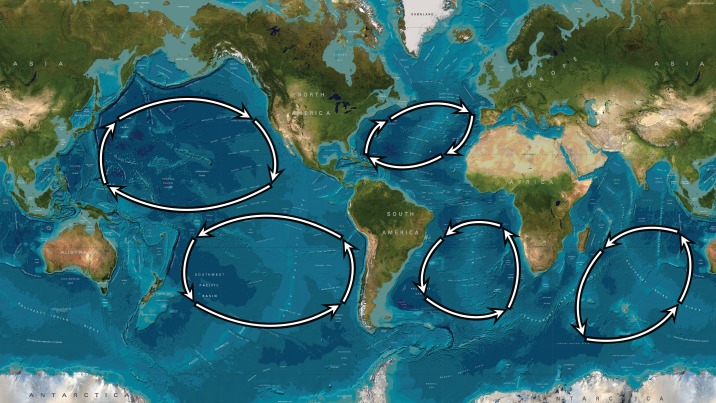
Sketch of the five most significant gyres on Earth, each centred at about 30^°^ north or south latitude in the subtropical regions, with the gyres in the Northern Hemisphere rotating clockwise, and those in the Southern Hemisphere rotating counter-clockwise. The North Pacific Gyre is the largest, but all five gyres have an immense size, covering ocean areas ofdiameters 4000–8000 km.

Many of the surface currents in our oceans link up to form gyres, which are large areas of water flowing in a roughly circular pattern and which tend to dominate the central regions of the open oceans; see figure 1 and the NASA web page http://oceanmotion.org/html/background/wind-driven-surface.htm. Of these, the largest are the gyres in the Pacific Ocean and in the Atlantic Ocean with, in addition, the Indian Ocean gyre; there are also a number of smaller gyres scattered across our oceans, e.g. the Beaufort Gyre in the Canada basin off the coast of Alaska (see [1]). The gyres in the Northern Hemisphere rotate predominantly clockwise, and those in the south, anticlockwise. The circulation of a gyre is controlled by three factors: the global wind patterns—the most significant contributor, the rotation of the Earth, and the presence of landmasses and bottom topography. They are, generally, very stable, with predictable properties and extent; indeed, floating debris often remains in a gyre for decades, and for that which reaches the centre, there is no escape. This occurs because a typical gyre circles around large areas—of the order of 10^6^ km^2^ in the case of the North Pacific Gyre—of essentially stationary, calm water. There is, however, considerable variation, from gyre to gyre, in the strength, width and effective depth of the surface currents that drive the gyre. Nevertheless, this general structure of a rotating flow, with little vertical motion, is the familiar pattern exhibited by a vortex, which is the most natural model to use as a basis for a mathematical discussion of these phenomena; e.g. [2]. So, for example, we might treat the gyre as a flow of constant or, possibly, zero vorticity; the eventual aim would be to combine regions of different vorticity in order to represent a specific gyre; however, in the initial phase of the work reported here, we will not pursue this detailed level of modelling. Although we might hope to describe, in a straightforward fashion, the underlying structure of smaller gyres using e.g. the *f*-plane approximation, we shall show, based on one simple and natural approximation, that we can begin the process of describing the structure of gyres of arbitrary size on the surface of a sphere.

Gyres are complex systems taken in their entirety, with many physical factors playing a role; they are, furthermore, significant drivers of the ‘ocean conveyor belt’. Such an important phenomenon encourages us to investigate how we might analyse them and discover their properties, particularly in the light of their surprisingly simple overall structure. On the one hand, we could tackle the problem as an exercise in modelling: use physical intuition and the available data to produce a representation of the flow (or, at the very least, some aspects of the flow). Such an approach is likely to be limited by what we suppose is important, and possibly by the requirement to formulate only that which can be analysed quite readily. On the other hand—and this is the classical applied mathematical philosophy—we start with the general equations that describe a fluid, identify the relevant scalings and parameters and see where that leads us. Of course, in either approach, it is necessary to make various overarching assumptions and sensible approximations, e.g. to work with a viscous or inviscid fluid, perhaps restrict to constant density, invoke the *f*- or *β*-plane approximation, ignore the turbulent nature of the real flow, and so on. Our starting premise is that the application of systematic applied mathematical techniques can make some useful contributions to studies in oceanography in general, and to the understanding of the structure of gyres in particular, even if this captures only the gross features. What is certain is that, with the applied mathematical approach, we can have considerable confidence in what may be uncovered. That gyres exist as a natural consequence of the general equations of motion (in spherical geometry), with clear and well-defined properties, is, we suggest, an important observation, even though a number of less critical physical characteristics may have to be suppressed in the development. We note that there is a growing interest in the study of ocean flows beyond the planar setting of the *f*- or *β*-plane approximation, within the framework of spherical geometry—see the discussion in [3] for a survey of the relevant research literature.

The main thrust of this work is to show how the Euler equation (coupled with the equation of mass conservation and the relevant boundary conditions) can lead directly to a solution that recovers the essential structure of a gyre of any size on the surface of the planet. Furthermore, the results allow, in principle, for any choice of velocity profile around the centre of the gyre (and so any observed variations from one gyre to another can be accommodated). We now outline the plan that we shall follow here. First, we present the governing equation of motion, and the equation of mass conservation, written in a rotating spherical coordinate system. This system is non-dimensionalized and scaled in order to produce a version that is relevant to a (relatively) thin layer of fluid on the surface of the Earth, whose dominant motion is purely rotational, so that any vertical motions are small (but still present). Then, we describe the resulting leading-order asymptotic solution which allows for any background vorticity and, at higher order, we indicate how this leads to a corresponding vertical structure in the flow field. However, it is the generality and accessibility of the leading-order problem that is the primary success of the work that we report here. In this model, we can have no surface stresses (wind) to drive the flow nor any decay mechanism—the fluid is inviscid—and so we must, perforce, allow the flow field to have been generated, and then this is to be maintained by a suitable pressure distribution at the free surface. Indeed, the pressure can be chosen in order to accommodate a (locally) non-flat surface, so that the observed domed structure in some gyres can be included. Only the assumption of a thin layer of fluid, which is naturally coupled with weak vertical motion, is needed in order to make headway; this is the problem that we describe in detail here.

## 2. Governing equations

**Figure 2.**
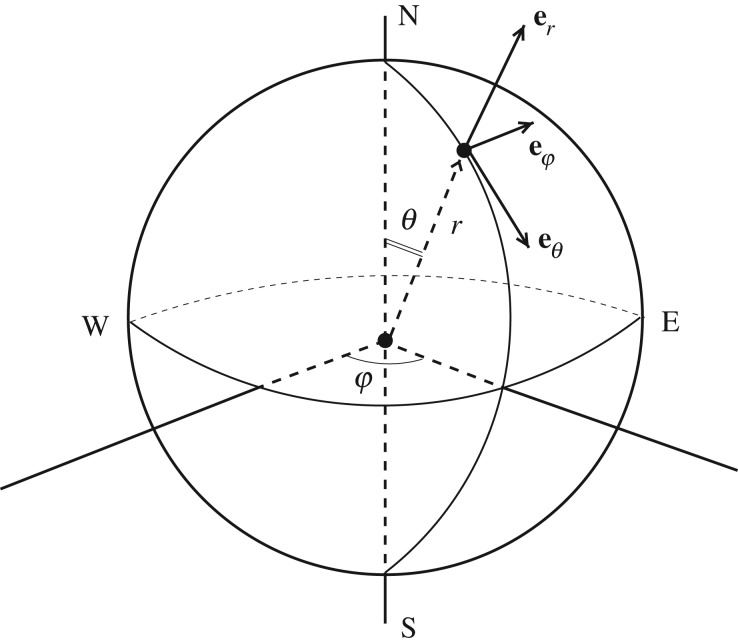
The spherical coordinate system, where *θ*∈[0,*π*] is the polar angle, *φ*∈ [0,2*π*) is the azimuthal angle and *r*′ is the distance from the origin: (*π*/2−*θ*) is the conventional angle of latitude, so that the North Pole corresponds to *θ*=0, the South Pole to *θ*=*π*, and the Equator to *θ*=*π*/2.

We introduce a set of (right-handed) spherical coordinates (*r*′,*θ*,*φ*): *r*′ is the distance (radius) from the centre of the sphere, *θ* (with 0≤*θ*≤*π*) is the polar angle (and then *π*/2−*θ* is conventionally the angle of latitude); *φ* (with 0≤*φ*<2*π*) is the azimuthal angle, i.e. the angle of longitude. (We use primes, throughout the formulation of the problem, to denote physical (dimensional) variables; these will be removed when we non-dimensionalize.) The unit vectors in this (*r*′,*θ*,*φ*)-system are (**e**_*r*_,**e**_*θ*_,**e**_*φ*_), respectively, and the corresponding velocity components are (*u*′,*v*′,*w*′); **e**_*φ*_ points from west to east, and **e**_*θ*_ from north to south (figure 2).

The Euler equation (describing an inviscid fluid) and the equation of mass conservation are

2.1 $$\begin{array}{rl} & \left(\frac{\partial }{\partial t^{\prime }}+u^{\prime }\frac{\partial }{\partial r^{\prime }}+\frac{v^{\prime }}{r^{\prime }}\frac{\partial }{\partial \theta }+\frac{w^{\prime }}{r^{\prime }\sin\theta }\frac{\partial }{\partial \varphi }\right)(u^{\prime },v^{\prime },w^{\prime })\\ & \;+\frac{1}{r^{\prime }}(-v^{\prime 2}-w^{\prime 2},u^{\prime }v^{\prime }-w^{\prime 2}\cot\theta ,u^{\prime }w^{\prime }+v^{\prime }w^{\prime }\cot\theta )\\ & \;=-\frac{1}{\rho^{\prime }}\left(\frac{\partial p^{\prime }}{\partial r^{\prime }},\frac{1}{r^{\prime }}\frac{\partial p^{\prime }}{\partial \theta },\frac{1}{r^{\prime }\sin\theta }\frac{\partial p^{\prime }}{\partial \varphi }\right)+(F_{r^{\prime }}^{\prime },F_{\theta }^{\prime },F_{\varphi }^{\prime }), \end{array}$$

and

2.2 $$\frac{1}{r^{\prime 2}}\frac{\partial }{\partial r^{\prime }}(r^{\prime 2}u^{\prime })+\frac{1}{r^{\prime }\sin\theta }\frac{\partial }{\partial \theta }(v^{\prime }\sin\theta )+\frac{1}{r^{\prime }\sin\theta }\frac{\partial w^{\prime }}{\partial \varphi }=0,$$

respectively, where *p*′(*r*′,*θ*,*φ*) is the pressure in the fluid, *ρ*′ is the density and (*F*_*r*′_′,*F*_*θ*_′,*F*_*φ*_′) is the body-force vector. In view of the particular structure that we shall describe here, the density is taken as constant (although an extension of this work can readily accommodate a fluid which has piecewise constant density with depth).

These equations describe the problem in a coordinate system with its origin at the centre of the sphere; we now associate (**e**_*r*_,**e**_*θ*_,**e**_*φ*_) with a point fixed on the sphere which is rotating about its polar axis. Thus, on the left of equation (2.1), we must introduce the additional terms

$$2\Omega^{\prime }\times \text{u'}\;\text{(Coriolis)}\;\text{and}\;\Omega^{\prime }\times (\Omega^{\prime }\times \text{r'})\;\text{(centripetal acceleration)},$$

where

$$\Omega^{\prime }=\Omega^{\prime }\;({\text{e}}_{r}\cos\theta -{\text{e}}_{\theta }\sin\theta ),\;\text{u'}=u^{\prime }{\text{e}}_{r}+v^{\prime }{\text{e}}_{\theta }+w^{\prime }{\text{e}}_{\varphi },\;\text{r'}=r^{\prime }\;{\text{e}}_{r},$$

with *Ω*′≈7.29×10^−5^ rad s^−1^, the constant rate of rotation of the Earth; these two contributions can be written as

2.3 $$2\Omega^{\prime }(-w^{\prime }\sin\theta ,-w^{\prime }\cos\theta ,u^{\prime }\sin\theta +v^{\prime }\cos\theta )-r^{\prime }\Omega^{\prime 2}(\sin^{2}\theta ,\sin\theta \cos\theta ,0).$$

The body-force vector, (*F*_*r*′_′,*F*_*θ*_′,*F*_*φ*_′), is chosen so that the component in the *r*′-direction is the familiar gravitational term (with *g*′=constant≈9.81 m s^−2^, constancy being reasonable for the depths of the oceans on the Earth); thus we write

2.4 $$(F_{r^{\prime }}^{\prime },F_{\theta }^{\prime },F_{\varphi }^{\prime })=(-g^{\prime },0,0).$$

At the free surface, *r*′=*R*′+*h*′(*θ*,*φ*), where *R*′≈6378 km is the (mean) radius of the Earth, we impose a surface pressure and the kinematic boundary condition

2.5 $$p^{\prime }=P_{s}^{\prime }(\theta ,\varphi )\;\text{on}\;r^{\prime }=R^{\prime }+h^{\prime }(\theta ,\varphi )$$

and

2.6 $$u^{\prime }=\frac{v^{\prime }}{r^{\prime }}\;\frac{\partial h^{\prime }}{\partial \theta }+\frac{w^{\prime }}{r^{\prime }\sin\theta }\;\frac{\partial h^{\prime }}{\partial \varphi }\;\text{on}\;r^{\prime }=R^{\prime }+h^{\prime }(\theta ,\varphi ),$$

respectively. At the bottom of the ocean, *r*′=*R*′+*d*′(*θ*,*φ*), which we take to be an impermeable, solid boundary, we have the corresponding kinematic condition

2.7 $$u^{\prime }=\frac{v^{\prime }}{r^{\prime }}\;\frac{\partial d^{\prime }}{\partial \theta }+\frac{w^{\prime }}{r^{\prime }\sin\theta }\;\frac{\partial d^{\prime }}{\partial \varphi }\;\text{on}\;r^{\prime }=R^{\prime }+d^{\prime }(\theta ,\varphi ).$$

## 3. The problem in spherical coordinates with a thin surface layer

We start the discussion of this aspect of the problem by defining a set of non-dimensional variables. To this end, we introduce the length scale *H*′ and a suitable speed scale, *c*′; the length scale is that associated with the average depth of the ocean, and so it is convenient to set

3.1 $$r^{\prime }=R^{\prime }+z^{\prime }.$$

The specific choice of speed scale is not critical to the analysis presented here (because of the particular limiting process that we invoke), so any suitable speed will suffice; for example, the average speed of currents that contribute to the motion of a gyre; the speed at the surface of the Earth, at the Equator, due to the rotation of the Earth about its axis; in the light of the hydrostatic pressure distribution (see (3.5) below), perhaps $c^{\prime }=\sqrt{H^{\prime }g^{\prime }}$. The original (dimensional) variables are now transformed, thereby producing the non-dimensional version of all the variables (no primes)

3.2 $$z^{\prime }=H^{\prime }z,\;(u^{\prime },v^{\prime },w^{\prime })=c^{\prime }(ku,v,w),\;p^{\prime }=\rho^{\prime }c^{\prime 2}p,$$

where we have used the constant density (*ρ*′) in the non-dimensionalization of the pressure; the scaling factor, *k*, associated with the vertical component of the velocity is yet to be chosen. On setting *ε*=*H*′/*R*′, the governing equations (2.1)–(2.2), for a steady flow, become

3.3 $$\begin{array}{rl} & \left(\frac{k}{\varepsilon }u\frac{\partial }{\partial z}+\frac{v}{1+\varepsilon z}\frac{\partial }{\partial \theta }+\frac{w}{(1+\varepsilon z)\sin\theta }\frac{\partial }{\partial \varphi }\right)(ku,v,w)\\ & \;+\frac{1}{1+\varepsilon z}(-v^{2}-w^{2},kuv-w^{2}\cot\theta ,kuw+vw\cot\theta )\\ & \;+2\frac{\Omega^{\prime }R^{\prime }}{c^{\prime }}(-w\sin\theta ,-w\cos\theta ,ku\sin\theta +v\cos\theta )\\ & \;-(1+\varepsilon z){\left(\frac{\Omega^{\prime }R^{\prime }}{c^{\prime }}\right)}^{2}(\sin^{2}\theta ,\sin\theta \cos\theta ,0)\\ & \;=-\left(\frac{1}{\varepsilon }\frac{\partial p}{\partial z},\frac{1}{1+\varepsilon z}\frac{\partial p}{\partial \theta },\frac{1}{(1+\varepsilon z)\sin\theta }\frac{\partial p}{\partial \varphi }\right)+\frac{R^{\prime }}{c^{\prime 2}}(-g^{\prime },0,0); \end{array}$$

and

3.4 $$\frac{k}{\varepsilon {\left(1+\varepsilon z\right)}^{2}}\frac{\partial }{\partial z}\{{\left(1+\varepsilon z\right)}^{2}u\}+\frac{1}{(1+\varepsilon z)\sin\theta }\left\{\frac{\partial }{\partial \theta }(v\sin\theta )+\frac{\partial w}{\partial \varphi }\right\}=0.$$

Now it is typical of thin-layer approximations—and quite obviously so for physically realistic flows—that the velocity component across the layer is small compared with the speeds along the layer. In the context of the motions observed in gyres, typical horizontal velocities are of the order of 0.01 m s^−1^ and the ratio of vertical speed to horizontal speed is about 10^−4^ to 10^−5^; see [4–7]. On this basis, we elect to examine the asymptotic structure of the problem on the assumption that the motion in the vertical direction is suitably weak. The most natural mathematical choice is given by *k*=*ε*^2^ (and we introduce the parameter *ω*=*Ω*′*R*′/*c*′, which is held fixed as *ε*→0, and so the full effects of rotation are retained). We have now introduced a characterization of the mathematical problem that we shall discuss; of course, it is not intended that the values of *ε* and *k* should match a particular pair encountered in the field. It is sufficient to formulate the problem that incorporates the property that *k* is smaller than *ε*, which is essentially what is observed. The corresponding equations and boundary conditions, all written in non-dimensional variables, with

3.5 $$p=P-\frac{H^{\prime }g^{\prime }}{c^{\prime 2}}z,$$

are therefore

3.6 $$\begin{array}{rl} & \left(\varepsilon u\frac{\partial }{\partial z}+\frac{v}{1+\varepsilon z}\frac{\partial }{\partial \theta }+\frac{w}{(1+\varepsilon z)\sin\theta }\frac{\partial }{\partial \varphi }\right)(\varepsilon^{3}u,v,w)\\ & \;+\frac{1}{1+\varepsilon z}(-\varepsilon v^{2}-\varepsilon w^{2},\varepsilon^{2}uv-w^{2}\cot\theta ,\varepsilon^{2}uw+vw\cot\theta )\\ & \;+2\omega (-\varepsilon w\sin\theta ,-w\cos\theta ,\varepsilon^{2}u\sin\theta +v\cos\theta )-(1+\varepsilon z)\omega^{2}(\varepsilon \sin^{2}\theta ,\sin\theta \cos\theta ,0)\\ & \;=-\left(\frac{\partial P}{\partial z},\frac{1}{1+\varepsilon z}\frac{\partial P}{\partial \theta },\frac{1}{(1+\varepsilon z)\sin\theta }\frac{\partial P}{\partial \varphi }\right); \end{array}$$

and

3.7 $$\frac{\varepsilon }{{\left(1+\varepsilon z\right)}^{2}}\frac{\partial }{\partial z}\{{\left(1+\varepsilon z\right)}^{2}u\}+\frac{1}{(1+\varepsilon z)\sin\theta }\left\{\frac{\partial }{\partial \theta }(v\sin\theta )+\frac{\partial w}{\partial \varphi }\right\}=0,$$

with

3.8 $$\begin{array}{rl} P & =P_{s}(\theta ,\varphi )+\frac{H^{\prime }g^{\prime }}{c^{\prime 2}}h\;\text{on}\;z=h(\theta ,\varphi ), \end{array}$$

3.9 $$\begin{array}{rl} \varepsilon u & =\frac{v}{1+\varepsilon h}\frac{\partial h}{\partial \theta }+\frac{w}{(1+\varepsilon h)\sin\theta }\frac{\partial h}{\partial \varphi }\;\text{on}\;z=h(\theta ,\varphi ), \end{array}$$

3.10 $$\begin{array}{rl} \;\text{and}\;\varepsilon u & =\frac{v}{1+\varepsilon d}\frac{\partial d}{\partial \theta }+\frac{w}{(1+\varepsilon d)\sin\theta }\frac{\partial d}{\partial \varphi }\;\text{on}\;z=d(\theta ,\varphi ), \end{array}$$

where we have also transformed (*h*′,*d*′)=*H*′(*h*,*d*), consistent with (3.2).

We set

3.11 $$P=\Pi -\frac{1}{4}\omega^{2}\cos(2\theta ),$$

and then the leading-order problem in *ε*, defined here by simply setting *ε*=0, is given by

3.12 $$\begin{array}{rl} & \frac{\partial \Pi }{\partial z}=0; \end{array}$$

3.13 $$\begin{array}{rl} & \left(v\frac{\partial }{\partial \theta }+\frac{w}{\sin\theta }\frac{\partial }{\partial \varphi }\right)v-w^{2}\cot\theta -2\omega w\cos\theta =-\frac{\partial \Pi }{\partial \theta }; \end{array}$$

3.14 $$\begin{array}{rl} & \left(v\frac{\partial }{\partial \theta }+\frac{w}{\sin\theta }\frac{\partial }{\partial \varphi }\right)w+vw\cot\theta +2\omega v\cos\theta =-\frac{1}{\sin\theta }\frac{\partial \Pi }{\partial \varphi }; \end{array}$$

3.15 $$\begin{array}{rl} \;\text{and}\; & \frac{\partial }{\partial \theta }(v\sin\theta )+\frac{\partial w}{\partial \varphi }=0. \end{array}$$

The boundary conditions appropriate at this order imply that we require

3.16 $$\Pi =P_{s}(\theta ,\varphi )+\frac{H^{\prime }g^{\prime }}{c^{\prime 2}}h+\frac{1}{4}\omega^{2}\cos(2\theta )\;\text{and}\;v\frac{\partial h}{\partial \theta }+\frac{w}{\sin\theta }\frac{\partial h}{\partial \varphi }=0\;\text{on}\;\;z=h(\theta ,\varphi );$$

the condition on the bottom is automatically satisfied with *d*(*θ*,*φ*)=constant, which is the simplification that we invoke in this first phase of the investigation. (We will make some further observations about the bottom boundary condition later.) It is now convenient to redefine the pressure at the surface so that it takes the form

3.17 $$\Pi =\Pi_{s}(\theta ,\varphi ),$$

which is regarded as the prescribed or chosen pressure that is to be associated with the flow (and corresponding surface distortion) that we describe here. At leading order, this is the pressure that must exist throughout the depth of the fluid layer, by virtue of equation (3.12) (but note that (3.5) shows the variation with depth associated with the hydrostatic pressure distribution); any additional variation of pressure with depth arises at the next order.

## 4. Solution of the leading-order problem

The main thrust of this analysis is to construct solutions, at this order, which recover any number of large flow patterns that mirror what is observed in our oceanic gyres. To see how this comes about, we use equation (3.15) to introduce the stream function *ψ*

4.1 $$v=\frac{1}{\sin\theta }\psi_{\varphi },\;w=-\psi_{\theta },$$

where subscripts denote partial derivatives; then, from (3.13) and (3.14), the compatibility condition generated by the elimination of *Π* produces the vorticity equation

4.2 $$\begin{array}{rl} & \psi_{\varphi }{\left(\frac{1}{\sin^{2}\theta }\psi_{\varphi \varphi }+\psi_{\theta }\cot\theta +\psi_{\theta \theta }-2\omega \cos\theta \right)}_{\theta }\\ & \;-\psi_{\theta }{\left(\frac{1}{\sin^{2}\theta }\psi_{\varphi \varphi }+\psi_{\theta }\cot\theta +\psi_{\theta \theta }-2\omega \cos\theta \right)}_{\varphi }=0. \end{array}$$

Here, the vorticity in the flow, at leading order, expressed in spherical coordinates, is

4.3 $$\nabla^{2}\psi =\frac{1}{\sin^{2}\theta }\psi_{\varphi \varphi }+\psi_{\theta }\cot\theta +\psi_{\theta \theta }.$$

The solution of equation (4.2) is constructed by first writing

4.4 ψ=−ωcos⁡θ+Ψ(θ,φ),

where *Ψ* is associated with the vorticity of the underlying motion of the ocean (relative to the Earth’s surface and not driven by the rotation of the Earth); equation (4.2) then becomes

4.5 $$\begin{array}{rl} & {\left(\Psi -\omega \cos\theta \right)}_{\varphi }{\left(\frac{1}{\sin^{2}\theta }\Psi_{\varphi \varphi }+\Psi_{\theta }\cot\theta +\Psi_{\theta \theta }\right)}_{\theta }\\ & \;-{\left(\Psi -\omega \cos\theta \right)}_{\theta }{\left(\frac{1}{\sin^{2}\theta }\Psi_{\varphi \varphi }+\Psi_{\theta }\cot\theta +\Psi_{\theta \theta }\right)}_{\varphi }=0. \end{array}$$

Throughout regions where ∇(Ψ−ωcos⁡θ)≠0, the rank theorem (see [8]) permits us to express the solution of (4.5) in the form F(Ψ−ωcos⁡θ)+2ωcos⁡θ=total vorticity, where

4.6 $$\frac{1}{\sin^{2}\theta }\Psi_{\varphi \varphi }+\Psi_{\theta }\cot\theta +\Psi_{\theta \theta }=F(\Psi -\omega \cos\theta )$$

and *F* is an arbitrary function; this is a new variant of the vorticity equation which, however, does not capture the solutions Ψ=ωcos⁡θ+A of (4.5), representing stationary flows *ψ*=*A*, where *A* is an arbitrary constant. (Note that the classical form of the vorticity equation is the familiar ∇^2^*Ψ*=*F*(*Ψ*).) The vorticity, which is aligned with the *r* coordinate, to leading order, therefore comprises two distinct components: that solely due to the rotation of the Earth ($\omega_{e}=2\omega \cos\theta$: ‘spin vorticity’) and that due to the motion of the ocean ($\omega_{s}=F(\Psi -\omega \cos\theta )$: ‘oceanic vorticity’), but this, we note, is coupled to the Earth’s rotation; the total vorticity *ω*_T_ is the sum of these two: *ω*_T_=*ω*_*e*_+*ω*_*s*_. Although one of these contributions (*ω*_*e*_) is completely prescribed, that associated with the movement of the ocean may be chosen to represent a particular gyre. We might expect that the vorticity inherent in the motion of the ocean is significantly larger than that provided by the rotation of the Earth; however, a suitable model for a gyre could be to allow parts of the flow field to be irrotational (oceanic vorticity *ω*_*s*_=0), and even for constant oceanic vorticity (*ω*_*s*_=*F*=constant) we see that there is no coupling.

We seek a solution of equation (4.6) in the form

**Figure 3.**
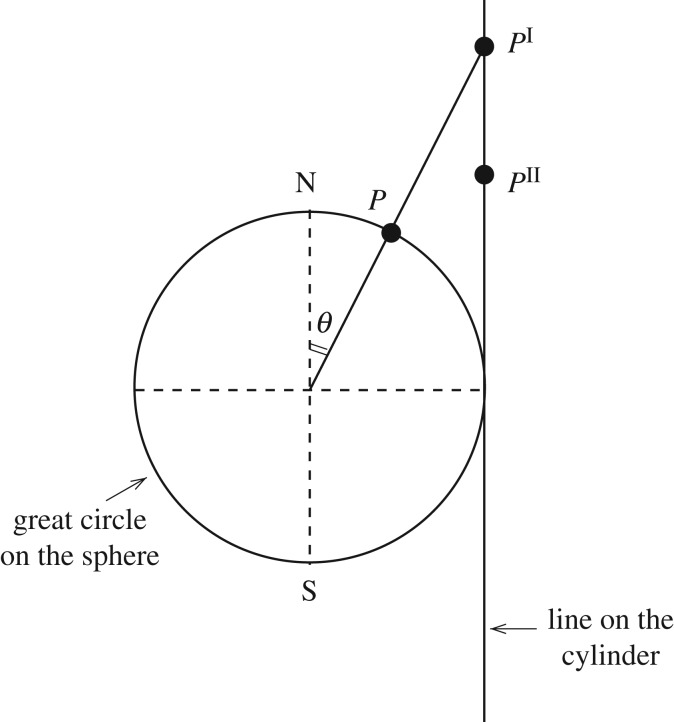
The Mercator projection maps a point $P(r^{\prime }\sin\theta \cos\varphi ,r^{\prime }\sin\theta \sin\varphi ,r^{\prime }\cos\theta )$ on the surface of the sphere, different from the two poles, to the planar point with Cartesian coordinates $\left(r^{\prime }\varphi ,r^{\prime }\ln[\tan(\theta /2)]\right)$. It is obtained by first mapping *P* to the intersection point $P^{\prime }(r^{\prime }\cos\varphi ,r^{\prime }\sin\varphi ,r^{\prime }\cot\theta )$ of the ray from the centre of the sphere to *P* with the cylinder tangent to the sphere along the Equator, then moving *P*′ vertically towards the equatorial plane to the point $P^{\prime \prime }(r^{\prime }\cos\varphi ,r^{\prime }\sin\varphi ,r^{\prime }\ln[\tan(\theta /2)])$ to bring about the same north–south length distortion rate as the east–west distortion rate (1/sin⁡θ) of the cylindrical projection, and subsequently unrolling the cylinder. The Mercator projection is conformal (preserves angles, transforming meridians into vertical lines and parallels into horizontal lines) but distorts areas, inflating them according to their distance from the equator, so that at the poles the scale becomes infinite (see [9]).

4.7 $$\Psi =\Psi (\xi ,\theta )\;\text{with}\;\xi =\varphi^{2}+{\left[A+\ln\left|\frac{1-\cos\theta }{\sin\theta }\right|\right]}^{2},$$

where *A* is an arbitrary constant; we observe that the logarithmic term here is that associated with the Mercator projection (figure 3) and with a Legendre function of the second kind; we define *φ* so that the centre of the gyre is always along *φ*=0. On using this transformation of variables, equation (4.6) becomes

4.8 $$\frac{4}{\sin^{2}\theta }(\Psi_{\xi }+\xi \Psi_{\xi \xi })+\frac{4}{\sin\theta }\left(A+\ln\left|\frac{1-\cos\theta }{\sin\theta }\right|\right)\Psi_{\xi \theta }+\Psi_{\theta }\cot\theta +\Psi_{\theta \theta }=F(\Psi -\omega \cos\theta ).$$

The general procedure, at this stage, is to choose or identify those functions *F* that are likely to be relevant or interesting or, at the very least, enable us to produce some simple, useful, closed-form solutions. We mention three cases that are readily accessible (although the third will not be pursued in this initial phase of the work).

The simplest case arises for zero oceanic vorticity, *F*=0; equation (4.8) then has a solution

4.9 Ψ=αln⁡ξ

where *α* is an arbitrary constant; this expression corresponds precisely with the classical solution for irrotational flow in two-dimensional, planar geometry. (We will describe the properties of this, and other solutions, in the next section.)

In order to examine the flow in a gyre in more detail, we should allow the (oceanic) flow to have some vorticity; the simplest choice is—quite obviously—constant vorticity (*F*=*γ*) and then a suitable solution is

4.10 Ψ=Dξ−γ ln⁡|sin⁡θ|−2D(|1−cos⁡θsin⁡θ|)2,

which can be written as

4.11 $$\Psi =\frac{\gamma }{\beta }\left[\varphi^{2}-{\left(A+\ln\left|\frac{1-\cos\theta }{\sin\theta }\right|\right)}^{2}-\beta \ln|\sin\theta |\right],$$

to within an additive constant, with *D*=*γ*/*β*; the strength of the velocity field is now proportional to *γ*/*β*, and the choice of *β* (for a given constant vorticity *γ*) controls the type of solutions available (as we will show in the next section).

In the above two examples, the oceanic-flow component uncouples from the flow that is driven by the rotation of the Earth; we now briefly mention an example where this is not the case. This more sophisticated choice involves setting *F* proportional to its argument, i.e. *F* is linear in (Ψ−ωcos⁡θ); we write equation (4.6)—the more natural choice, as will immediately become clear—in the form

4.12 $$\frac{1}{\sin^{2}\theta }\Psi_{\varphi \varphi }+\Psi_{\theta }\cot\theta +\Psi_{\theta \theta }=\lambda (\Psi -\omega \cos\theta ),$$

where λ is an arbitrary constant. This equation can be solved by using, for example, spherical harmonic functions; this is a routine but rather tiresome procedure, and not developed here. We do, however, make one observation: a particular integral of equation (4.12) is

$$\Psi =\frac{\lambda \omega }{\lambda +2}\cos\theta ,$$

and so there exists a solution of the homogeneous equation (for λ=−2) which is resonant with this particular rotation-driven solution. This appears to be worthy of further investigation.

Finally, we observe that there is a choice of oceanic vorticity which ensures that the total vorticity (*ω*_T_) is zero; this requires F(Ψ−ωcos⁡θ)=−2ωcos⁡θ, which corresponds to the choice

$$F(\varphi )=2\omega \tanh\left(\frac{2\varphi }{\omega }\right).$$

If we ignore the planetary (spin) vorticity (by setting *ω*=0), equation (4.6) becomes the familiar equation describing stationary vortex structures in an ideal fluid; see ([10], ch. 5) for a systematic analysis of its exact solutions and for a detailed discussion and graphical illustration of their qualitative features. The presence of planetary vorticity in equation (4.6) is geophysically relevant and it also alters considerably the underlying mathematical structure of the problem due to the intricate coupling between the oceanic and the planetary vorticity components. This new equation brings with it some exciting prospects for future investigations. Some general observations about the existence and nature of its solutions are given in appendix A.

## 5. Some properties and examples of the leading-order solution

In order to examine the relevance of the solutions presented in the preceding section, we must develop some detailed properties of them. Most particularly, we must confirm that solutions do exist which describe the rotational motion evident in gyres. The first, and most illuminating property, is the streamline pattern generated by our choices of stream function; these provide confirmation that we have found solutions that correspond to large gyres.

**Figure 4.**
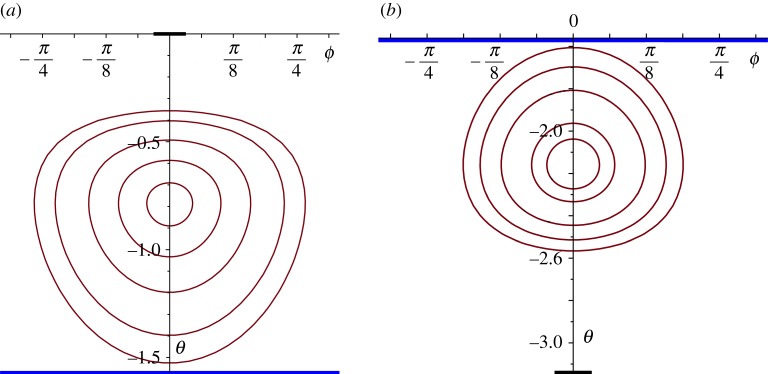
Streamlines for an irrotational flow in the Northern Hemisphere ((*a*), with the centre of the gyre at *π*/4) and in the Southern Hemisphere ((*b*), with the centre of the gyre at 11*π*/16); the blue line indicates the Equator and the centre of the heavy black lines, the two poles.

**Figure 5.**
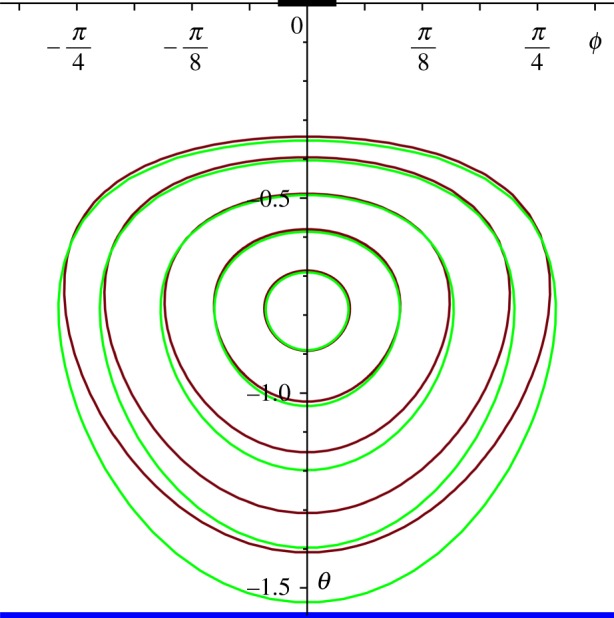
Streamlines with rotation effect included, for $\omega /\alpha =\frac{1}{2}$, in red; corresponding streamlines without rotation effect shown in green (for the case presented in figure 4*a*, Northern Hemisphere).

The simplest case arises from the choice of an irrotational flow; some typical streamlines (defined by *ξ*=constant, for a given *A*; see (4.7)) for two such flows (*A*=0) are shown in figure 4. (All the figures that we present show the streamlines plotted in (*θ*,*φ*)-coordinates, but represented in the (flat) Cartesian plane; the ‘physical’ streamlines are the corresponding curves in (*θ*,*φ*)-coordinates on the surface of the sphere; note that *θ* is measured downwards, north to south, from zero in these figures.) Figure 4 shows the streamlines associated with the ‘oceanic’ part of the flow field, i.e. based solely on the stream function given in (4.9); the streamlines as seen on the surface of the ocean, however, are generated by the stream function *ψ*, given in (4.4). Certainly, we must expect that the shape of these streamlines is dominated by the term in *Ψ* because if ωcos⁡θ, the term due solely to the rotation of the Earth, was the dominant term, this would correspond to streamlines *θ*=constant. In figure 5, we show the effects of including this term at a moderate level ($\omega /\alpha =\frac{1}{2}$); the streamlines are compared with those that correspond to the flow shown in figure 4 (no spin vorticity), the distortion being evident but not overwhelming.

**Figure 6.**
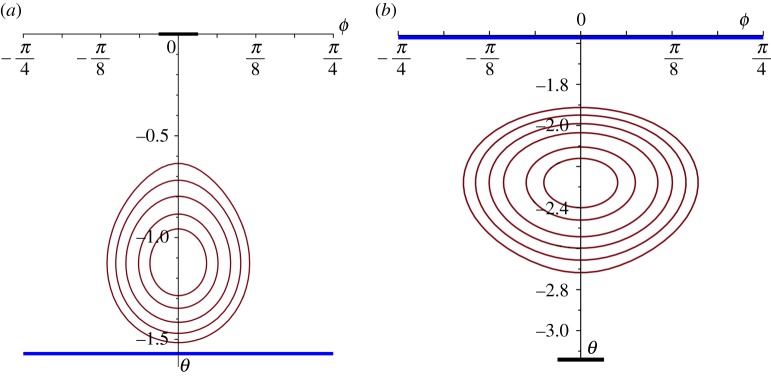
Streamlines for a constant vorticity flow in the Northern Hemisphere ((*a*), *A*=−0.4, *β*=4) and in the Southern Hemisphere ((*b*), *A*=1.5, *β*=7); the regions of the gyre depicted here are restricted by a bounding streamline; the blue line indicates the Equator and the centre of the heavy black lines, the two Poles.

**Figure 7.**
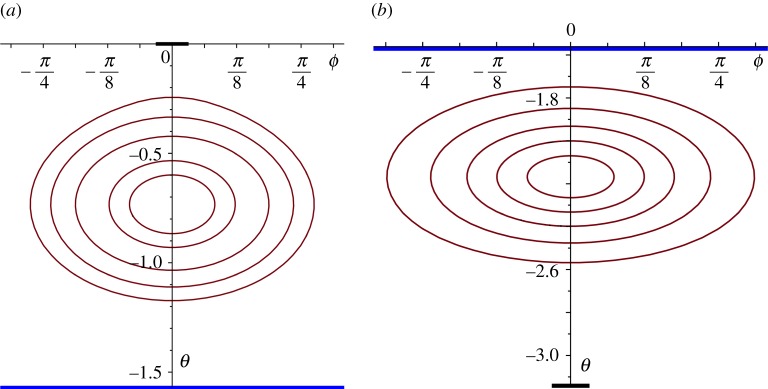
Streamlines for a constant vorticity flow in the Northern Hemisphere ((*a*), *A*=−3.5, *β*=12) and in the Southern Hemisphere ((*b*), *A*=3.3, *β*=14); the regions of the gyre depicted here are restricted by a bounding streamline; the blue line indicates the Equator and the centre of the heavy black lines the two poles.

In the case of constant-vorticity flow, we have—not surprisingly—a more complex structure; see (4.11). For *β*≤2, there exist solutions with closed streamlines, but these necessarily extend across the whole globe north-to-south (0<*θ*<*π*), unless we arbitrarily restrict the region of applicability; there are also solutions with open streamlines. An altogether more satisfactory situation arises for *β*>2; closed streamlines exist and, for a range of values of *A* and *β*, there are two families: one in the Northern Hemisphere and one in the Southern, the switch from one to the other requiring a change of sign in *A*. Some of these solutions do extend across the Equator (for the choice *A*=0, for example)—so we reject these as not being relevant in this context—but for larger *β*s and suitable *A*, we find many solutions that are restricted to just one hemisphere and, furthermore, their extent is limited by a bounding streamline; two examples are shown in figure 6. These two families are necessarily limited by the Equator, across which neither exist; again, we could arbitrarily limit the region of applicability to ensure that the gyre stops some distance short of the Equator or, more satisfactorily, choose the parameters so that the bounding streamline is positioned as we wish it (as we have done here). Indeed, this is precisely the model we would choose to use when we combine together regions of different vorticity: perhaps, an outer region of constant vorticity and an inner one that is irrotational or, possibly, stationary. As the parameter *β* increases, so more variation is possible in the shape of the gyre; again, with a bounding streamline that ensures the gyre sits wholly within one hemisphere, we show another two solutions (figure 7). It is evident that there is considerable freedom available within the structure of these solutions, allowing the possibility of producing models for gyres that extend over considerable regions of the oceans and which correspond, roughly, with the overall shapes that are observed. The four examples shown in figures 6 and 7 indicate quite clearly how the shape and the position of the gyre can be adjusted within one hemisphere. (All these streamline patterns will be (slightly) distorted by the inclusion of the spin contribution, as shown for the irrotational case in figure 5).

On the basis of the explicit solutions that we have obtained for the streamlines (and so for the associated velocity field), we can determine the pressure distribution that is required to maintain the motions described here. In the case of the irrotational flow, represented by the stream function (4.9), the pressure, *Π*(*θ*,*φ*) (see (3.11)), is readily obtained; from this we can find the pressure prescribed at the surface (see the first equation in (3.16)) in the region where the streamlines exist

5.1 $$P_{s}(\theta ,\varphi )=-2\frac{\left\{\alpha \omega [A+\ln|(1-\cos\theta )/\sin\theta |]+\alpha^{2}/\sin^{2}\theta \right\}}{\varphi^{2}+{\left[A+\ln|(1-\cos\theta )/\sin\theta |\right]}^{2}}-\frac{H^{\prime }g^{\prime }}{c^{\prime 2}}h(\theta ,\varphi ),$$

to within an arbitrary additive constant. The free surface, *z*=*h*(*θ*,*φ*), is an arbitrary function of the stream function, *ψ*: this automatically satisfies the second equation in (3.16). We may therefore choose *h* to accommodate any observed surface profiles (such as those with a domed structure), and (5.1) then gives the surface-pressure distribution necessary to maintain the flow and the shape of the surface. The corresponding result for constant oceanic vorticity is

5.2 $$\begin{array}{rl} P_{s}(\theta ,\varphi ) & =-{\left(\frac{\gamma }{\beta }\right)}^{2}\left\{\frac{1}{2\sin^{2}\theta }{\left[\beta \cos\theta +2\ln\left(\frac{1-\cos\theta }{\sin\theta }\right)+2A\right]}^{2}+\beta {\left[A+\ln\left(\frac{1-\cos\theta }{\sin\theta }\right)\right]}^{2}\right\}\\ & \;-\gamma^{2}\ln(\sin\theta )+\frac{2\omega \gamma }{\beta }\ln\left(\frac{1-\cos\theta }{\sin\theta }\right)-\varphi^{2}{\left(\frac{\gamma }{\beta }\right)}^{2}\left(\frac{2}{\sin^{2}\theta }-\beta \right)-\frac{H^{\prime }g^{\prime }}{c^{\prime 2}}h(\theta ,\varphi ), \end{array}$$

to within an arbitrary additive constant, where *h* is again an arbitrary function of *ψ*.

## 6. Some observations concerning the solution at the next order

The structure of the governing equations, and associated boundary conditions, (3.6)–(3.10), indicates that it is reasonable to seek an asymptotic solution based on the asymptotic sequence {*ε*^*n*^}_*n*≥0_. Indeed, to some extent, this guided our choice of parameter dependence that describes the velocity component in the *z*-direction: *k*=*ε*^2^. On this basis, we seek a solution expressed in the form

$$q(\theta ,\varphi ,z;\varepsilon )∼\sum_{n=0}^{\infty }\varepsilon^{n}q_{n}(\theta ,\varphi ,z),\;\varepsilon \to 0,$$

where *q* represents each of the unknowns *u*, *v*, *w*, *P*, *h* (although, in the case of *h*, the dependence on *z* is omitted). Thus, the solution designated by the zero subscript is precisely that obtained in §4. (We will provide some comments concerning the validity of this asymptotic solution later.) The system of equations and boundary conditions that describe the *O*(*ε*) problem are

6.1 $$\begin{array}{rl} & v_{0}^{2}+w_{0}^{2}+2\omega w_{0}\sin\theta +\omega^{2}\sin^{2}\theta =P_{1z}, \end{array}$$

6.2 $$\begin{array}{rl} & {\left(v_{0}v_{1}\right)}_{\theta }+\frac{w_{0}v_{1\varphi }+w_{1}v_{0\varphi }}{\sin\theta }-2w_{0}w_{1}\cot\theta -2\omega (w_{1}+zw_{0})\cos\theta -\omega^{2}z\sin(2\theta )=-P_{1\theta }, \end{array}$$

6.3 $$\begin{array}{rl} & v_{0}w_{1\theta }+v_{1}w_{0\theta }+\frac{{\left(w_{0}w_{1}\right)}_{\varphi }}{\sin\theta }+(v_{0}w_{1}+v_{1}w_{0})\cot\theta +2\omega (v_{1}+zv_{0})\cos\theta =-\frac{1}{\sin\theta }P_{1\varphi }, \end{array}$$

6.4 $$\begin{array}{rl} \;\text{and}\; & u_{0z}+\frac{{\left(v_{1}\sin\theta \right)}_{\theta }+w_{1\varphi }}{\sin\theta }=0, \end{array}$$

with

6.5 $$\begin{array}{rl} P_{1} & =P_{1s}+\left(\frac{H^{\prime }g^{\prime }}{c^{\prime 2}}\right)h_{1}\;\text{on}\;z=h_{0}(\theta ,\varphi ), \end{array}$$

6.6 $$\begin{array}{rl} u_{0} & =v_{0}h_{1\theta }+v_{1}h_{0\theta }+\frac{w_{0}h_{1\varphi }+w_{1}h_{0\varphi }}{\sin\theta }\;\text{on}\;z=h_{0}(\theta ,\varphi ) \end{array}$$

6.7 $$\begin{array}{rl} \;\text{and}\; & u_{0}=0\;\text{on}\;z=d. \end{array}$$

In this formulation of the problem that describes the perturbation of the solution found earlier, we have invoked a Taylor expansion about *z*=*h*_0_(*θ*,*φ*) in the surface boundary conditions; this is a permitted procedure here because, as is already evident, the problem is polynomial in *z* (which is the typical behaviour in the shallow-water approximation). The inclusion of both *h*_1_ and *P*_1*s*_ is to allow some freedom (hopefully leading to a suitable simplification) in the small correction terms to the surface profile and the surface pressure, respectively.

From equation (6.1), and the associated boundary condition (6.5), we obtain the correction to the pressure

$$P_{1}=(v_{0}^{2}+w_{0}^{2}+2\omega w_{0}\sin\theta +\omega^{2}\sin^{2}\theta )(z-h_{0})+P_{1s}+\left(\frac{H^{\prime }g^{\prime }}{c^{\prime 2}}\right)h_{1},$$

which provides a contribution to the hydrostatic pressure distribution. We could, at a later stage, make choices relating to the (small) adjustments to both the surface profile and the surface pressure distribution. However, an obvious route, allowing some simplification, is to choose the relation between *h*_1_ and *P*_1*s*_ so that pressure perturbation takes the form

6.8 $$P_{1}=(v_{0}^{2}+w_{0}^{2}+2\omega w_{0}\sin\theta +\omega^{2}\sin^{2}\theta )z.$$

It then follows that there is a solution of equations (6.2)–(6.3), for *v*_1_ and *w*_1_, for which these two functions are proportional to *z*; we set

6.9 $$v_{1}=zV_{1}(\theta ,\varphi ),\;w_{1}=zW_{1}(\theta ,\varphi ),$$

and then, from (6.4) and (6.7), we obtain

6.10 $$u_{0}=\frac{{\left(V_{1}\sin\theta \right)}_{\theta }+W_{1\varphi }}{\sin\theta }(d^{2}-z^{2}).$$

In this development, *u*_0_ at the surface is known (from (6.10)), and then the kinematic condition, (6.6), is satisfied by a suitable choice of *h*_1_(*θ*,*φ*); this, with the choice leading to (6.8), fixes the pressure perturbation at the surface. The problem providing the construction of solutions for *V*_1_ and *W*_1_, associated with appropriate choices for the background flow (represented by *Ψ*), can be formulated, although explicit, closed-form, solutions of the underlying second-order partial differential equation, for general *Ψ*, cannot be written down (although this is possible for the case of irrotational flow). Nevertheless, our outline demonstrates the essential character of the problem at this next order, involving a polynomial structure in *z* (and no obvious non-uniformities exist provided that the gyres sit strictly within a hemisphere, and not too close to a pole or the Equator). Furthermore, with this additional information, we are able to provide a representation of the way in which the direction of the horizontal component of the velocity vector varies with depth; this is controlled, at this order of approximation, by

$$\frac{v_{0}(\theta ,\varphi )+\varepsilon zV_{1}(\theta ,\varphi )}{w_{0}(\theta ,\varphi )+\varepsilon zW_{1}(\theta ,\varphi )}$$

although the details will depend on how all these functions depend on *θ* and *φ*.

## 7. Discussion

The ideas presented here have demonstrated, based on Euler’s equation with vorticity included, that models for gyres can be constructed. This has been possible by invoking a thin-layer—equivalently a shallow-water—approximation, and nothing more. This particular asymptotic interpretation has the benefit of generating a problem, at leading order, which is both nonlinear and in a rotating, spherical coordinate system. Furthermore, this system can be solved in terms of the underlying vorticity and, in some cases, explicit, simple solutions can be obtained. It is clear, however, that the success of this approach is down to the exclusion of any variation in the vertical coordinate at leading order; this is, of course, precisely the hallmark of classical shallow-water theory. Indeed, consistent with this, the structure in the vertical (*z*) direction is also classical: polynomial in *z*. Because gyres are predominantly circulatory in motion, with very weak vertical motion (as confirmed in field data), we submit that we have generated a useful and relevant model. The difficulties encountered at higher order (i.e. being unable to construct simple, closed-form solutions) are certainly irritating, but this aspect is relatively insignificant when set against the type of flows available—and there are many possibilities—in our leading-order solution. This is the main thrust of the work presented here.

The structure of gyres, as generated by our vorticity equation (for the oceanic component of the flow field), (4.6), can take many and varied forms, the extent of the possibilities is, we suggest, worthy of further investigation. We have presented some examples of the streamlines that describe the oceanic component, both for irrotational flow and constant vorticity. (One intriguing and unlooked for ingredient is the appearance—the natural choice when solving—of a transformation of coordinates that involves the essential component of the Mercator projection; see (4.7).) In the case of irrotational flow (given by (4.9)), the flow can be centred anywhere on the surface of the sphere (by adjusting the constant *A*), and extends in closed curves around this centre; to be physically relevant and appropriate, the region of the flow must be restricted. This ensures that the gyre can be allocated to one of the hemispheres, and outside this region we presumably impose a different type of flow field (which could be no motion at all). It is gratifying to obtain streamlines that correspond, in their general shape, to the flow configuration of gyres, but the irrotational case is far from satisfactory. Firstly, there is no natural boundary to the extent of such a flow (and we might hope and expect this to be the case for a gyre); secondly, we might reasonably argue that, in any event, the gyres are not likely to be irrotational structures. Either there is some appropriate vorticity distribution (within some regions of our oceans), or the flow is essentially stationary. So although it is reasonable to note the existence of solutions for irrotational flow, it would seem that they do not provide useful models for gyres.

On the other hand, flows with constant (oceanic) vorticity offer far better prospects. For sufficiently large *β*—indeed, for *β*>2—and then for suitable *A* (see (4.11)), there exist solutions that sit in only one hemisphere and which have a bounding streamline (so there is no question of arbitrarily truncating the region). As *β* and *A* are varied, so the centre of the gyre, the shape of the streamlines and the extent of the region all change. We have not carried out a comprehensive examination of exactly how *β* and *A* affect the properties of the gyre, so this is something else for future study. We have, however, explored the nature of the problem sufficiently to confirm the general picture described above, and to produce a number of examples.

It is clear, using our description of the underlying flow (as implied by equations (3.12)–(3.16)), that we must have some oceanic vorticity, for otherwise the streamlines would be simply *θ*=constant, which do not represent gyres. Because we may, in principle, assign any vorticity to the flow, and we have no mechanism in our model for initiating or driving the gyre, we must choose the sign of the vorticity to match the observed clockwise (Northern Hemisphere) or counter-clockwise (Southern Hemisphere) rotations. This fixes the overall flow structure; in addition, we may impose any free-surface profile, by selecting a suitable *h*(*ψ*). Then, in order to maintain this motion (and surface), we require a surface-pressure distribution; this we may regard as a modelling of—and a replacement for—the effects of pressure and wind-action at the surface; see (5.1) and (5.2).

We believe that our approach to a better understanding of some of the underlying principles and properties of gyres is, within the confines of an inviscid theory, built on a firm foundation. The asymptotic method used here—the shallow-water approximation—that leads to the basic flow patterns that we have described, is the most natural one in this context, and it ensures that all the relevant contributions are retained. However, what we describe here can be no more than the first stage in an investigation that must, we suggest, be taken further. There is a lot of freedom afforded by our formulation of the problem, and so there are many avenues that need to be explored; we mention some of the more obvious ones.

Clearly, it would be wise to seek solutions associated with other choices of vorticity, and one obvious candidate here is to examine how expansions in spherical harmonics might play a role. With other solutions available (and this also applies to the zero and constant vorticity examples found here), we should investigate how regions of different vorticities can be combined; this is not a simple exercise. The difficulty arises because, based on what we have discovered so far, different vorticity distributions lead to different shapes for the streamlines; cf. the classical Rankine vortex, for which all streamlines are circles with a common centre. It is therefore impossible, across a streamline, to switch from one vorticity to another. We will need some mechanism for producing a transition between two sets of streamlines associated with two different vorticity distributions. The only possibility, in general, is to allow a region of no flow (stationary conditions) between the two areas of different vorticity; but perhaps other special flows can be discovered which overcome this difficulty. As our results currently stand, we can certainly have a constant vorticity flow and introduce a stationary flow both inside and outside two specific streamlines. (The former would correspond to the stagnant areas observed in the central regions of some of our gyres.) Related to this, the new type of vorticity equation, (4.6), certainly needs a detailed investigation, which might throw some light on the choices of vorticity distribution that are allowed and reasonable; a few initial observations have been provided in appendix A.

It is evident that some work needs to be done on the higher-order terms in our asymptotic solution. In a more complete discussion, we should aim to develop a fairly comprehensive description of the *O*(*ε*) term (which we have managed to do in the irrotational case, this being reduced to a quadrature; the complexity of the resulting solution, and its questionable relevance to the observed motions of gyres, persuaded us not to reproduce it here). With a clearer idea of the structure of the *O*(*ε*) solution, we can then attempt some general observations about the higher-order terms. We can report that our experience with this problem suggests that the asymptotic expansion is uniformly valid provided that the flows are not too close to the poles or the Equator; the polynomial behaviour in *z* precludes any difficulties in this variable.

All the comments thus far relate, more-or-less, to aspects of the model and the approach adopted here. However, we should consider whether there are any reasonably accessible avenues that might lead to an improvement in the model. One area of significance, that is relevant to gyres that we observe, is the role of the topography (both undersea and the surrounding land masses). Indeed, it is generally accepted that these are fundamental to the definition of the regions occupied by gyres. The solution presented earlier used the simplest bottom boundary condition: *d*=constant; but our formulation admits a more general choice. As with the surface kinematic condition (see the comments after (5.1)), we may introduce a bottom topography that is described by *d*=*d*(*ψ*). A viable model, within our scenario, is to introduce precisely this form of bottom condition, and to regard this as the primary driver for the geometric constraints on the gyre. We could then argue that this leads to the description of the flow field in terms of our stream function, with the associated structure of the free surface: *h*=*h*(*ψ*). Of course, to accommodate other behaviours of the bottom, we would need to specify appropriate functions *d*(*θ*,*φ*); see (2.7) and (3.10). However, in order to progress the general approach that we have developed here, we cannot arbitrarily assign *d*(*θ*,*φ*). Nevertheless, it might be possible to use the *method of multiple scales*, and introduce a depth variation (which could be large, to the extent of allowing for the appearance of shores and land masses) but which varies *slowly* in *θ* and *φ*, e.g. *d*=*d*(*εθ*,*εφ*), or something similar. Such a possibility needs further investigation.

In conclusion, we have demonstrated that conventional ideas of fluid mechanics, with associated standard approximation procedures, can make a contribution to the study, and understanding, of gyres. As we have made clear, this can only be the start: there are many avenues that need considerably more work done on them, involving deeper and more extensive examination.

## Figures and Tables

**Figure 8.**
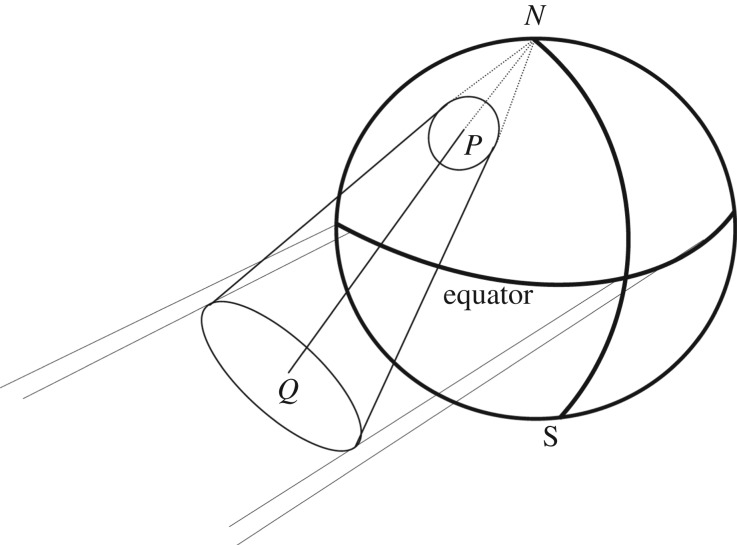
Schematic illustration of the stereographic projection from the sphere to the complex plane, mapping the point *P* on the sphere with the North Pole *N* excised to the intersection point *Q* of the equatorial plane with the ray from *N* to *P*, and the spherical surface region $O^{\prime }$ around *P* onto the planar region O around *Q*. While the stereographic projection distorts areas dramatically, it preserves angles (it is conformal) and circularity (it transforms all circles on the sphere to circles on the plane, apart from the circles passing through the North Pole, which are transformed into lines).

## Data accessibility

All data are provided in full in the paper.

## Appendix A. Some general considerations about the nonlinear problem for *Ψ*

Outside the realm of explicit solutions to (4.6) one can expect to identify key qualitative features of the flow dynamics from specific properties of the vorticity *F* by using techniques from the theory of elliptic partial differential equations, as described below. To gain further insight, one can rely on approaches specific to Lagrangian coherent structures (see [11]), a direction of research that we plan to develop in the near future.

Let us perform the stereographic projection of the unit sphere centred at at origin, from the North Pole to the equatorial plane (figure 8):

A 1 $$\xi =r\;e^{i\phi }\;\text{with}\;r=\cot\left(\frac{\theta }{2}\right)=\frac{\sin\theta }{1-\cos\theta }.$$

where (*r*,*ϕ*) are the polar coordinates in the equatorial plane. We have

$$\cos\theta =\frac{\xi \bar{\xi }-1}{\xi \bar{\xi }+1},\;\sin\theta =\frac{\sqrt{\xi \bar{\xi }}}{\xi \bar{\xi }+1},\;\partial_{\theta }=-\frac{\xi }{\sin\theta }\partial_{\xi }-\frac{\bar{\xi }}{\sin\theta }\partial_{\bar{\xi }},\;\partial_{\phi }=i\xi \partial_{\xi }-i\bar{\xi }\partial_{\bar{\xi }}.$$

After several cancellations one can see that equation (4.6) takes the form

A 2 $$\Psi_{\xi \bar{\xi }}=\frac{F(\Psi -\omega ((\xi \bar{\xi }-1)/(\xi \bar{\xi }+1))}{{\left(1+\xi \bar{\xi }\right)}^{2}}.$$

For constant vorticity (*F*=*γ*), the general solution of (7.2) is seen to be

A 3 $$\Psi (\xi ,\bar{\xi })=\gamma \ln(1+\xi \bar{\xi })+\Phi (\xi ,\bar{\xi }),$$

where *Φ* is an arbitrary harmonic function. Note that the solution (4.10) is among the family (7.3).

It is convenient to use (4.4) to write (7.2) equivalently as

$$\psi_{\xi \bar{\xi }}+2\omega \frac{1-\xi \bar{\xi }}{{\left(1+\xi \bar{\xi }\right)}^{3}}-\frac{F(\psi )}{{\left(1+\xi \bar{\xi }\right)}^{2}}=0.$$

or, with the understanding that (*x*,*y*) are the Cartesian coordinates in the complex *ξ*-plane, as the semi-linear elliptic equation

A 4 $$\Delta \psi +8\omega \frac{1-(x^{2}+y^{2})}{{\left(1+x^{2}+y^{2}\right)}^{3}}-\frac{4F(\psi )}{{\left(1+x^{2}+y^{2}\right)}^{2}}=0,$$

where $\Delta =\partial_{x}^{2}+\partial_{y}^{2}$ is the Laplace operator. At the ocean surface, a gyre is bounded by a level set of the stream function, say *ψ*=0, with the flow at the surface $O^{\prime }$ of the gyre determined in the new coordinates by the solution to (7.4) inside the planar region O, being the stereographic projection of $O^{\prime }$ (figure 8). Consequently we have to solve (7.4) in O with Dirichlet boundary data

A 5 ψ=0on ∂O.

Let us mention some relevant analytic approaches for the study of the semi-linear elliptic boundary-value problem (7.4)–(7.5).

(i) *Existence of solutions for a given smooth vorticity* *F* *in a specified bounded region* O *with smooth boundary* ∂O: Writing the lower-order part of (7.4) as F(x,y,ψ), we say that a smooth function *ψ*^±^, defined on an open planar set whose interior comprises O∪∂O, is a sub/super-solution if $\pm [\Delta \psi^{\pm }+F(x,y,\psi^{\pm })]\leq 0$ throughout O. For example, if *F*(0)≥0, then *ψ*^+^≡0 is a supersolution in the Northern Hemisphere as (7.1) yields *x*^2^+*y*^2^>1. On the other hand, if *F*(0)≤0, then *ψ*^−^≡0 is a sub-solution in the Southern Hemisphere because (7.1) now ensures *x*^2^+*y*^2^<1; more sophisticated sub/super-solutions are provided by the functions *ψ* associated with the special solutions (4.9) and (4.11), or by seeking radial functions (in which case one has to solve a second-order ordinary differential equation). If *F* is smooth and if one can find sub/super-solutions *ψ*^±^ such that *ψ*^−^≤*ψ*^+^ throughout O and such that *ψ*^−^≤0≤*ψ*^+^ on ∂O, then a functional-analytic approach (see [12]) shows that there exists a solution *ψ* to (7.4) such that *ψ*^−^≤*ψ*≤*ψ*^+^ throughout O.
(ii) *The comparison method and uniqueness*: If the function *F* is non-decreasing, using the strong maximum principle (see [13]), we can reduce the above requirements that *ψ*^−^≤*ψ*^+^ throughout O and *ψ*^−^≤0≤*ψ*^+^ on ∂O to merely *ψ*^−^≤0≤*ψ*^+^ on ∂O by taking advantage of the comparison principle: if *ψ* is a solution and *ψ*^+^ a supersolution with 0≤*ψ*^+^ on ∂O, then the mean-value theorem yields
  $$0\leq \Delta (\psi -\psi^{+})-\frac{4[F(\psi )-F(\psi^{+})]}{{\left(1+x^{2}+y^{2}\right)}^{2}}=\Delta (\psi -\psi^{+})-\frac{4F^{\prime }(\tilde{\psi })}{{\left(1+x^{2}+y^{2}\right)}^{2}}(\psi -\psi^{+}),$$
  and the strong maximum principle ensures *ψ*≤*ψ*^+^ in O, if this inequality holds on ∂O; a similar comparison can be made with a sub-solution. In particular, in this setting, we therefore have uniqueness. The above approach is also valid without the monotonicity assumption on *F*, but only if the domain O is sufficiently narrow or of sufficiently small area—see [14]. Moreover, if there exist constants *M*>0 and *q*≥1 with
  A 6 $$|F(s)|\leq M(1+|s{|}^{q}),\;s\in R,$$
  then the solution between the sub- and super-solution is a local minimum of the functional
  A 7 $$E(\psi )=\frac{1}{2}\int_{O}|\nabla \psi {|}^{2}\;dx\;dy-\int_{O}\left[8\omega \frac{1-(x^{2}+y^{2})}{{\left(1+x^{2}+y^{2}\right)}^{3}}\psi -\frac{4F(\psi )}{{\left(1+x^{2}+y^{2}\right)}^{2}}\right]dx\;dy,$$
  where $F(t)=\int_{0}^{t}F(s)\;ds$; see [12].
(iii) *Numerical methods*. The variational formulation (7.7) permits the implementation of the finite-element method, providing a versatile computational approach towards gaining quantitative and qualitative insight, but one can also use finite-difference methods—see the discussion in [15].
